# Assessing the toxicity of green *Agaricus bisporus*-based Cadmium Sulfide nanoparticles on *Musca domestica* as a biological model

**DOI:** 10.1038/s41598-024-70060-y

**Published:** 2024-09-14

**Authors:** Hanan I. Elhenawy, Noura A. Toto, Abdelazeem S. Eltaweil, Hussein K. Hussein, Maria Augustyniak, Lamia M. El-Samad

**Affiliations:** 1 Department of Zoology, Faculty of Science, Alexandria University, Alexandria, Egypt; 2 Department of Zoology, Faculty of Science, Damanhur University, Damanhur, Egypt; 3 Department of Engineering, College of Engineering and Technology, University of Technology and Applied Sciences, Ibra, Sultanate of Oman; 4 Chemistry Department, Faculty of Science, Alexandria University, Alexandria, Egypt; 5 Institute of Biology, Biotechnology and Environmental Protection, Faculty of Natural Sciences, University of Silesia in Katowice, Bankowa 9, 40-007 Katowice, Poland

**Keywords:** *Musca domestica*, *Agaricus bisporus*, Cadmium Sulfide, Cell apoptosis, HSP70 expression, Biochemical components, Cell biology, Ecology, Ecology, Environmental sciences

## Abstract

The common housefly, *Musca domestica*, known for transmitting over 100 infections, was studied using green-synthesized Cadmium Sulfide nanoparticles (CdS NPs) from *Agaricus bisporus*. These CdS NPs were tested on third-instar larvae under laboratory conditions using dipping and feeding methods with concentrations (75, 100, 125, 150, 175, and 200 µg/mL). The toxicity, measured by LC50, was found to be 138 µg/mL for dipping treatment and 123 µg/mL for feeding treatment. Analysis with an energy-dispersive X-ray microanalyzer confirmed Cd accumulation in the larval midgut, indicating penetration of CdS NPs into the organism, which may potentially increase their toxicity. CdS NPs caused disruptions in Heat Shock Protein 70, cell apoptosis, and various biochemical components. Scanning electron microscopy revealed morphological abnormalities in larvae, pupae, and adults exposed to CdS NPs. Ultrastructural examination showed significant midgut tissue abnormalities in larvae treated with 123 µg/mL of CdS NPs. Our study demonstrated that green-synthesized CdS NPs from *A. bisporus* can effectively control the development of *M. domestica* larvae.

## Introduction

*Musca domestica* L., scientifically classified under Diptera: Muscidae is a globally distributed species of Diptera commonly found in diverse human and animal communities worldwide^1,2^. Due to its close association with human activity, *M. domestica* poses significant concerns for public health^3^. For example, house flies can act as vectors for viral, bacterial, parasitic, and zoonotic pathogens^4^. They are active diurnally, causing annoyance and facilitating the spread of pathogens^5^. *Musca domestica* is responsible for transporting pathogenic organisms into the human diet from filthy environments such as sewage and garbage^6^. This transmission can occur through mechanical means, including contamination by the fly's external body parts or through the regurgitation and defecation that may happen after the flies consume the food^7^.

To combat houseflies, insecticides have traditionally been employed as the most common method, with chemical compounds such as organophosphates^8^, chlorinated hydrocarbons, pyrethroids, and carbamates being utilized for the past several decades^9,10^. The detrimental environmental effects and significant costs associated with chemical insecticides have prompted entomologists to explore alternative, eco-friendly, and cost-effective methods for controlling houseflies^1^. Therefore, researchers have recognized the need to shift away from chemical insecticides and have emphasized the importance of utilizing natural pesticides to combat housefly infestations^11^.

Biopesticides, derived from various organisms such as fungi, bacteria, nematodes, algae, or natural products, play a crucial role in numerous applications, making them a vital component in the continuous exploration of innovative formulations^12,13^. Biopesticides, produced through biological synthesis with plant extracts and microbes, have stability, environmental compatibility, and affordability advantages. These nanopesticides can be manufactured without high-energy processes or hazardous substances, making them highly desirable^14^. Many green synthesized NPs have been utilized for biomedical and technological applications such as Ag, FeO_3_, ZnO, and Au NPs^15–19^. Green-synthesized nanoparticles that control house flies demonstrate superior effectiveness to pesticides while being more cost-effective, biodegradable, and posing minimal risks to humans and the environment^20,21^. Biological synthesis methods for nanoparticles offer distinct advantages, as they are often simpler and more cost-effective compared to traditional methods^22^.

In the last decade, using nanomaterials in pest management has offered significant advantages from their properties, helping reduce dependence on traditional insecticides and mitigating resistance development^23^. Incorporating nanomaterials, which vary from 1 to 100 nm, into pest management strategies can lead to more rapid and effective reactions against pests, surpassing the effectiveness of traditional-sized particles^24,25^. Nanoparticles possess extraordinary physicochemical characteristics owing to their minute dimensions and specific arrangement, allowing them exceptional adaptability across various applications^26^.

Myconanotechnology, an emerging field in nanotechnology, explores the use of fungi for the eco-friendly green synthesis of nanomaterials^27^. Ongoing research in myconanotechnology has led to the discovery of new applications, such as pest control and the prevention of bacterial and fungal diseases^28^. Various types of macrofungi, including *Ganoderma spp*., *Pleurotus spp*., *Lentinus spp*., *Agaricus bisporus*, and *Lentinus spp*., have been successfully employed for the synthesis of metal nanoparticles such as platinum, silver, cadmium, iron, and gold. These metal nanoparticles synthesized through myconanotechnology have shown great potential in advancing both industrial and biomedical sectors^29,30^

The *Agaricus bisporus*, commonly known as the button mushroom, is a white-colored fungus highly regarded for its delicious taste and nutritional benefits^31^. Adding supplements to the growth substrates can enhance the nutritional value of *A. bisporus* mushrooms^32^. Researchers have reported the utilization of mushroom extracts derived from *A. bisporus* for their ability to reduce metal ions and synthesize metal nanoparticles^33,34^. Among various green metal-based nanoparticles, Cadmium Sulphide (CdS) nanoparticles have garnered attention due to their multifunctional properties, including antibacterial, insecticidal, and anticancer characteristics, which arise from their unique electrical conductivity, chemical reactivity, and exceptional strength^35,36^.

Green-synthesized CdS nanoparticles (CdS NPs) offer several advantages over traditional materials, primarily due to their unique properties and environmentally friendly production methods^37^. Unlike conventional methods, which often involve toxic chemicals, the green synthesis of CdS NPs using *Agaricus bisporus* eliminates the use of harmful substances, reducing environmental impact and production costs^29^.

The nanoparticles' small size and high surface area enhance their interaction with biological systems, leading to significant morphological deformities and elevated mortality in the target organisms^38^. Additionally, the biogenic synthesis process leverages natural extracts, ensuring that the nanoparticles are guided by biomolecules like proteins and enzymes, which contributes to their unique shapes and functionalities^39^. This method not only makes the CdS NPs cost-effective and sustainable but also highlights their potential for broader applications in various environmental and industrial contexts^40^.

Comprehensive studies are still necessary to assess the impact of Cd-containing nanoparticles on various life history features. This includes both short- and long-term physiological impacts at both proteomic and genomic levels, occurring during and after exposure^41^. Exposure to Cd during the larval stage has been found to adversely affect survival, development time, and overall adult organisms' survival rate^42^. The interaction of Cd with biological macromolecules might interfere with the typical cell division process, leading to adverse effects on the growth and development of the organism^43^. Additionally, nanoparticles (NPs) have an extensive ability to penetrate cell membranes^44^.

Insect cell-based Cd toxicity assays have emphasized the crucial role of Heat Shock Protein 70 (HSP70) in protecting cells against Cd-induced damage^45^. Additionally, the energy in an insect's body, representing the total energy derived from glycogen, proteins, lipids, and glucose, can indirectly reflect overall energy reserves. Various factors, such as pesticide exposure, can influence the energy available to insects^46^.

The research targeted to investigate the insecticidal properties of environmentally friendly multifunctional CdS NPs^47^ against the third instar larvae (L3) of *M. domestica*, serving as a representative model organism. To assess the insecticidal activity, we utilized two methods—dipping and feeding—and tested a sequence of increasing concentrations of CdS NPs. Morphological and ultrastructural changes in larvae were evaluated using progressive imaging techniques, including Scanning Electron Microscopy (SEM) and Transmission Electron Microscopy (TEM). Furthermore, the detrimental effects were determined by assessing stress in the insect's body by measuring cell viability, the expression level of HSP70, and biochemical analyses like glycogen, sugar, lipid, and protein levels.

## Materials and methods

### *Agaricus bisporus* sourse

For the synthesis of CdS-NPs, fresh Agaricus bisporus obtained from a local market in Alexandria, Egypt, served as the primary botanical source and transferred to Alexandria University's chemistry research laboratory. Cadmium chloride monohydrate (CdCl_2_.H_2_O, 99.995%, CAS No. 654054-66-7), sodium sulfide. nonahydrate (Na2S.9H_2_O, ≥ 98%, CAS No. 1313-84-4), were purchased from Sigma-Aldrich. Ethanol(C_2_H_5_OH) with a purity of 99% was obtained from Mumbai (India).

### Synthesis of CdS NPs

The *Agaricus bisporus* was initially meticulously washed, cut into small fragments, and subjected to a 10-h drying period in an 80 °C oven. Then, the resulting dried mushroom residues were ground into a fine powder using a blender. Subsequently, 5 g of this powdered material were mixed with 75 mL of deionized water in a beaker. This mixture was heated with continuous stirring for 1 h and allowed to cool to room temperature, after which it was filtered to yield the final extract employed for the synthesis. In the next phase, a 50 mL solution of cadmium chloride (0.1 M) was prepared, and 5 mL of the filtered extract was introduced into this solution. Sequentially, a 50 mL solution of sodium sulfide (0.1 M) was slowly added drop by drop until the solution's color transitioned from light brown to orange. The solution was then stirred for 16 h at temperatures ranging from 60 °C to 80 °C in a dark environment^48,49^. The purification step was achieved by centrifugation of the colloidal solution, and the resulting precipitate was collected and subjected to multiple washes with distilled water and ethanol. Finally, the washed precipitate was dried overnight in an oven set at 60 °C, culminating in the successful synthesis of CdS NPs. The prepared CdS NPs were characterized using different tools, as mentioned in the supplementary information (Text S1).

#### Insect sampling

Adult *M. domestica* flies were captured using a bait jar in El-Behaira and transferred to Alexandria University's Entomology research laboratory. They were bred in 30 cm × 30 cm × 30 cm entomological cages at approximately 26 °C, 70% humidity, and a consistent 12-h light and 12-h dark cycle. Adult houseflies received an equal-volume mixture of table milk and sugar for water and food. Oviposition was facilitated on a plastic plate with a 1:3 weight ratio mixture of bran and milk (100 g)^50,51^. Housefly larvae were nourished in plastic cups with a nutrient medium including 5 g yeast, 9 g powdered milk, and 100 g fine bran mixed in 100 ml water^52^.

### Experimental design

Dipping and feeding methods were used to assess the larval bioassay.

In the dipping method, The larvicidal potential of the green CdS NPs concentrations was assessed using the dipping method, according to Aziz^53^). The larvicidal potential of varying concentrations of green CdS NPs was evaluated by dipping 20 third instar larvae in six CdS NPs concentrations (75, 100, 125, 150, 175, and 200 µg/mL) for 30 s. Subsequently, the larvae were transferred to filter paper inside a plastic cup, with distilled water used as a control. After 30 s, larvae were placed in a container containing wheat bran, yeast, water, and dry milk powder mixture (1:1:1 ratio). Mortality was recorded 24 h later, and LC_50_ was determined. The experiment was conducted in controlled lab conditions (26 ± 1 °C, 14:10 light: dark cycle, 70% humidity), with each dose replicated three times.

In the feeding method, the larvicidal effect of synthesized green CdS NPs was evaluated using the standard procedures described eslewhere^52^. Third-instar *M. domestica* larvae were exposed to green CdS NPs in concentrations ranging from 75 to 200 µg/mL. Three plastic cups, with 20 mL of simulated diet, were prepared for each concentration. The experiment included three replicates for each concentration, 20 individuals in each cup, resulting in a total of 60 larvae per concentration. Larval mortality was recorded 24 h post-exposure, using distilled water as the control. The LC_50_ was calculated to determine the lethal concentration.

Following treatment through either the feeding or dipping method, the larvae were monitored until they reached the pupal stage. The pupae were placed in small plastic cups under controlled lab conditions (26 ± 1 °C, 14:10 light: dark cycle, 70% humidity). Subsequently, a pupal bioassay was carried out after 24 h to evaluate any abnormalities that may have occurred in the pupae.

### Nanotoxic analysis of CdS NPs on larvae

#### X-ray Detection of Cd and S in *M. domestica* larval tissues

Cd and S concentrations in *M. domestica* third instar larval midgut tissues were assessed using SEM analysis. The Jeol JSM-5300 instrument in Tokyo, Japan, with a 20 kV accelerating voltage, examined three samples from each group. SEM analysis occurred at Alexandria University's Faculty of Science in Egypt, equipped with a Link-Isis EDX for element peak identification, done automatically by the EDX software through correlation analysis^54^.

#### Using flow cytometry to assess cell viability

Following the manufacturer's instructions, apoptosis in the larvae was evaluated using the TACSTM Annexin-V-FITC apoptosis detection kit (TASC Annexin-V-FITC, Catalogue Number: TA4638, Germany). To make a cell suspension, the samples were homogenized at 4 °C in cold phosphate-buffered saline (PBS, pH 7.4). After collection, the cells were washed twice with PBS before being resuspended in 195 µL of binding buffer. Adding 5 µL of the annexin-V-FITC conjugate reagent to the cell suspensions, they were incubated for 10 min in the dark. The cells were then cleaned and resuspended in 190 µL of binding buffer with an addition of 10 µL of propidium iodide solution. Becton Dickinson's flow cytometry system in Franklin Lakes, New Jersey, USA, was used to assess various cell states. The collected data was processed using Becton Dickinson's Cell Quest Pro software, version 5.2.1, 2005 (San Jose, CA, USA). Apoptotic and non-apoptotic properties of different cell populations were identified by analyzing the fluorescence signals from propidium iodide and Annexin-V-FITC^55,56^.

#### Assessment of HSP70 gene expression

Three larval midgut tissues of *M. domestica* were chosen randomly for total RNA extraction, following the manufacturer's instructions for the TRIzolTM Plus RNA Purification Kit (Invitrogen, USA). The spectrophotometer and agarose gel electrophoresis were used to assess the quality and purity of the recovered RNA. The relative expression levels of HSP70 in the larval midgut tissues were investigated through a two-step RT-PCR method. The High-Capacity cDNA Reverse Transcription Kit (Applied Biosystems, Catalogue Numbers 4368813) was used as the PCR template to reverse transcribe the RNA isolated from the larvae into cDNA. The RT-qPCR reactions employed specific forward (F) and reverse (R) primers for HSP70 (F: 5′-TACCCCCTTGTCTTTGGGTATTGAAACC-3′, R: 5′-GGTCTGGGTTTGCTTAGTGGGGATTG-3′). The PCR procedure comprised two minutes of initial denaturation at 94 °C, 25 cycles of 30 s at 94 °C, 30 s at 55 °C, 1 min at 72 °C, and a final extension for 10 min at 72 °C. PCR standardization was conducted using β-actin-specific primers (actin F: 5′-CACGCCATCCTGCGTCTGGA-3′ and actin R: 5′-CCACATCTGCTGGAAGGTGG-3′), following the protocol outlined by^57^. Using the Maxima SYBR Green/ROX qPCR Master Mix (2X) kit (Thermo-scientific, USA), real-time PCR analysis was carried out in the protocol described by^58^. The effects of CdS NPs on glycogen, sugar, lipid contents, and protein levels were examined, as mentioned in the supplementary information (Text S2).

#### SEM and TEM analysis

To analyze third-instar larvae, pupa, and adult samples of *M. domestica*, we followed the SEM procedure outlined by Toto et al.^50^). Specimens were fixed using a 4F:1G solution (pH 7.2, 0.1 M phosphate buffer) following Section 5.3 guidelines, involving three hours at a cold temperature and an extra two hours at 4 °C in 2% osmium tetroxide. Subsequently, samples underwent a two-hour cleaning in phosphate-buffered saline (PBS) at 4 °C before SEM examination. Dehydration involved sequential 15-min immersions in increasing ethanol concentrations. Specimens were mounted on aluminum stubs for SEM analysis and, for enhanced conductivity, coated with gold–palladium using a sputter-coating unit (JFC-1100 E). The SEM examined microstructural characteristics and captured comprehensive photographs^59^.

For TEM analysis of *M. domestica* third larval instar, specimens were fixed in a 2.5% glutaraldehyde solution (pH 7.4, 0.1 M cacodylate buffer) for 24 h. After fixation, samples underwent a series of ethanol and acetone increases for drying and were embedded in Araldite and Epon. Thin sections were cut using an LKB Bromma ultramicrotome (2088 Ultrotome, Mississippi, USA), stained with lead citrate and uranyl acetate, and examined with an 80 kV JEOL 100CX electron microscope, capturing images on the camera^60,61^.

### Statistical analysis

Kaplan–Meier survival testing determined mortality curves for all groups. The log-rank test (Chi^2^, *p* < 0.05) assessed significant variances between curves. LC_50_ values for different concentrations were calculated using the AAT Bioquest® online calculator at https://www.aatbio.com/tools/ec50-calculator^62^. Pupal inhibition percentages were calculated using IBM Corp package version 20.0 (Armonk, NY). Chi-square and Fisher's exact tests compared categorical variables, with Fisher's exact test correcting for chi-square when > 20% of cells had an expected count < 5. Quantitative data were presented as numbers and percentages. Significance was assessed at the 5% level. Cell viability data underwent one-way ANOVA^2,63^. Gene expression analysis, targeting the relative expression value for HSP70, was performed using a t-test^64^. All statistical analyses were executed using Statistica MatlabR2023a 13.3 software.

## Results

### Characterization of CdS NPs

**Figure 1:**
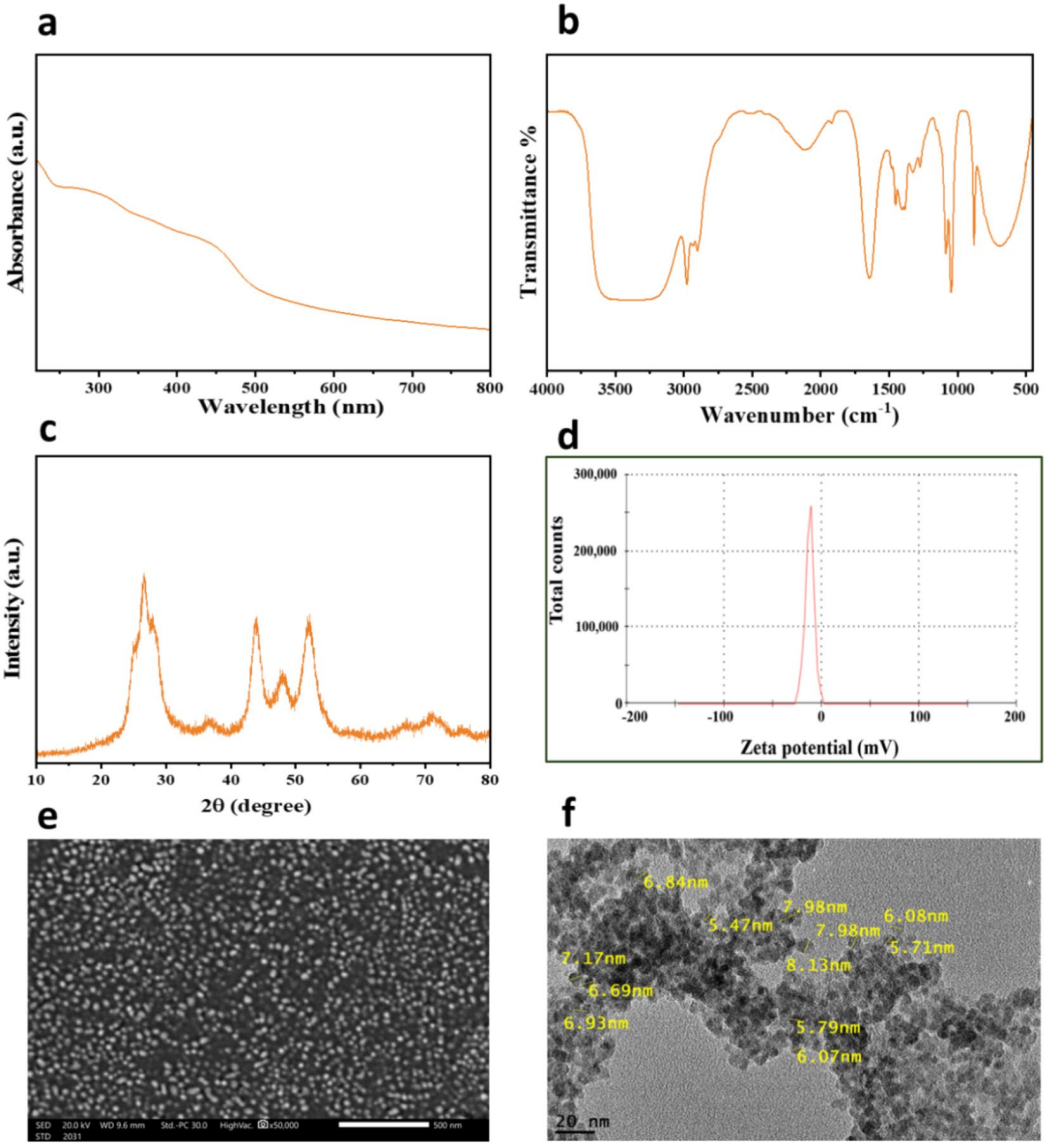
CdS NPs: UV–Vis full spectrum (**a**), FTIR spectrum (**b**), XRD pattern (**c**), zeta potential analysis (**d**), SEM image (**e**), and TEM image (**f**) of CdS NPs.

The UV–VIS absorption spectrum for CdS NPs is illustrated in Fig. 1. Within this spectrum, the CdS NPs display an absorption band edge at approximately 480 nm, which is notably blue-shifted when compared to micrometer-sized CdS particles typically commencing at around 550 nm. This prominent blue shift, signifying an augmented band gap, can be attributed to the quantum size effect. Based on these results, the estimated average diameter of these CdS nanoparticles confined within the pore channels is expected to be less than 10 nm^65^.

**Figure 2:**
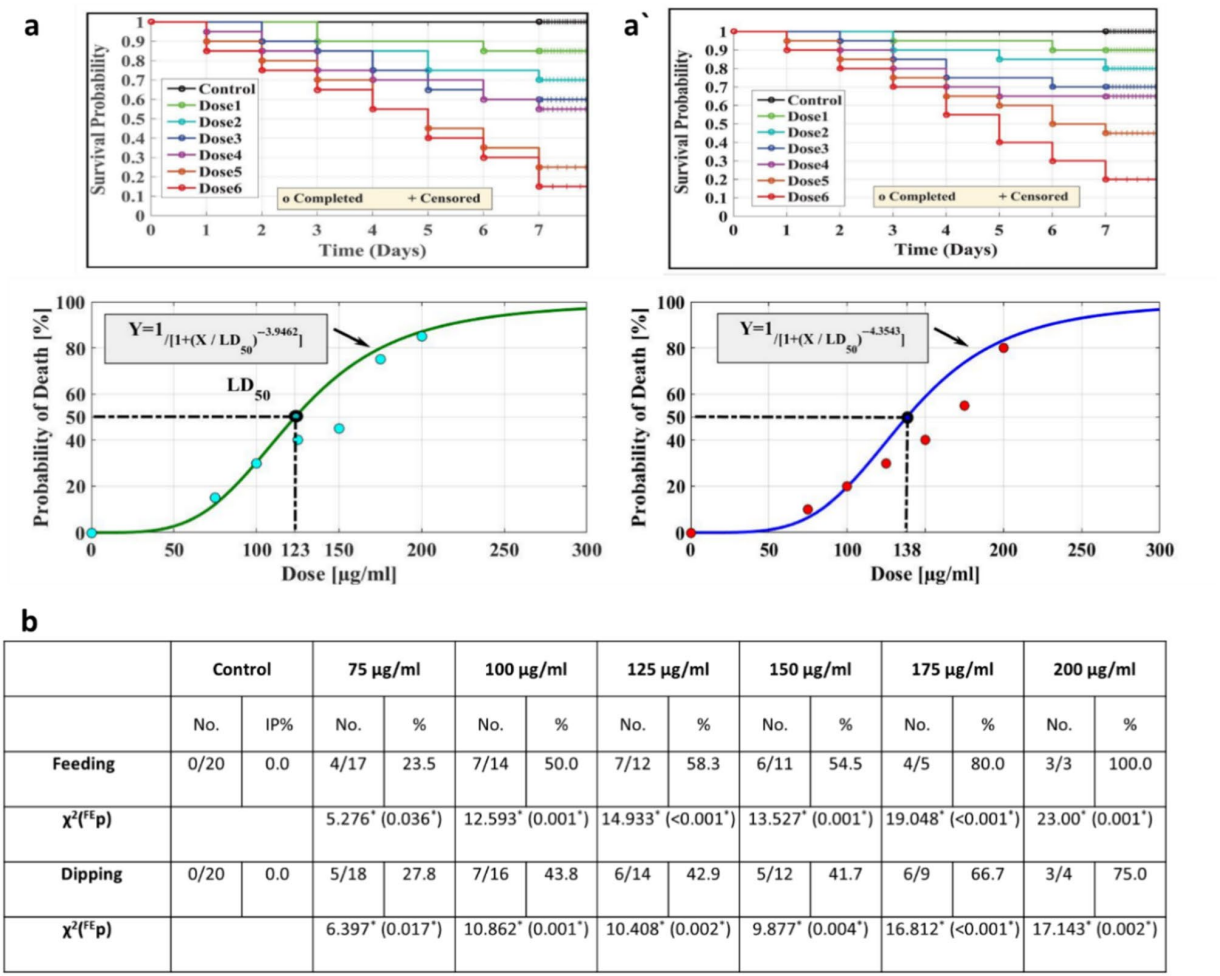
Kaplan–Meier survival analysis for larvae treated with CdS NPs at the following doses 1 ÷ 6: 75, 100, 125, 150, 175, and 200 µg/mL, respectively, by (**a**) feeding method with LC50 123 μg/mL. (**a'**) the dipping method with LC50 138 μg/mL. Pupal inhibition percentages (**b**) of *M. domestica* resulting from CdS NP treatment using feeding and dipping methods. (Abbreviations: χ^2^—Chi-square test, FE—Fisher Exact, p—*p* value for comparing the studied groups, and *—Statistically significant at *p* ≤ 0.05).

FTIR spectra were employed for the identification of main functional groups in CdS NPs (Fig. 1b). The spectrum of CdS (Fig. 2c) revealed a sharp peak at 1642.5 cm^−1^, attributed to the vibration of the carbonyl stretching of amide-I in protein molecules from mushroom plants^66^. Furthermore, the peak at 1399.6 cm^−1^ was assigned to O–C=O asymmetric stretching vibrations^67^. The band within the 1000–1120 cm^−1^ range was associated with stretching vibrations of the sulfate group, and the peak at 685.9 cm^−1^ was indicative of Cd–S stretching^68^. Moreover, the peak at 2895 cm^−1^ is related to aliphatic hydrocarbons C–H stretching which could be originated from the hydrocarbons in the extract. The broad peak in 3350 cm^−1^ represents hydroxyl groups in the structure.

The XRD pattern of CdS NPs (Fig. 1c) revealed distinct peaks at 2θ values of 24.9°, 26.7°, 28.1°, 36.4°, 44.0°, 48.0°, 52.0°, 66.7°, 71.1°, and 75.8°, corresponding to the (100), (002), (101), (102), (110), (103), (203), (211), and (105) crystalline planes of the hexagonal structure of CdS^69^. The observed broadening of these peaks suggests the presence of residual plant constituents inherent to the biogenic synthesis process. Notably, the XRD patterns did not reveal any detectable peaks indicative of impurities, confirming the high purity of the CdS NPs. The stability of the synthesized CdS NPs was evaluated through zeta potential measurements (Fig. 1d), yielding a noteworthy value of -19.5. This negative zeta potential for CdS NPs indicates a robust repulsive force among the prepared CdS NPs, suggesting excellent stability and hindering aggregation within CdS NPs colloidal solution. The synthesized nanomaterials' morphology and particle size characteristics were examined using a transmission electron microscope (TEM) and scanning electron microscopy (SEM). In the SEM images of CdS NPs (Fig. 1e), spherical particles were observed to coexist, forming some aggregations. However, TEM images (Fig. 1f) provided a more detailed analysis, confirming the aggregation of CdS NPs with an average particle size ranging from 5 to 8 nm. This size range aligns well with the results obtained from the UV–Vis spectrum, reinforcing the consistency and accuracy of the characterization methods applied to CdS NPs.

### Feeding method

*M. domestica* larvae were exposed to CdS NPs in concentrations (75, 100, 125, 150, 175, and 200 µg/mL) as part of their daily diet. Higher concentrations led to a significant increase in cumulative mortality in the third larval instar compared to the control group. Kaplan–Meier survival analysis (Fig. 2a) depicted the anticipated survival time. The AAT Bioquest® calculator determined larval mortality in the lowest and highest concentrations, yielding an LC_50_ of 123 μg/mL. Mortality increased proportionally with CdS NP concentrations. Through the feeding strategy, larvae absorbed NPs through ingestion and direct contact, combining surface contact and ingestion as NP uptake strategies.

### Dipping method

The effects of dipping *M. domestica* L3 in various doses of CdS NPs (75, 100, 125, 150, 175, and 200 µg/mL) were depicted by the mortality curve in (Fig. 2a'). Kaplan–Meier survival analysis revealed that CdS NPs exerted an opposing, dose-dependent influence on insect survival. At 125 and 150 µg/mL, the mortality rate decreased for both treated and normal larvae. The AAT Bioquest® calculator yielded an LC_50_ value of 138 µg/mL.

### Pupicidal method

The impact of CdS NPs on the *M. domestica* pupal stage was assessed using feeding and dipping experiments, as presented in (Fig. 2b). The results indicate substantial toxicity of CdS NPs toward the pupal stage. Pupation inhibition (IP%) in the feeding assay ranged from 0% at the lowest concentration to 100% at the highest concentration. In the dipping assay, pupation inhibition varied from 0% at the lowest concentration to 75% at the highest concentration.

### Quantification of CdS NPs accumulated in the whole body of *M. domestica* larvae

**Figure 3:**
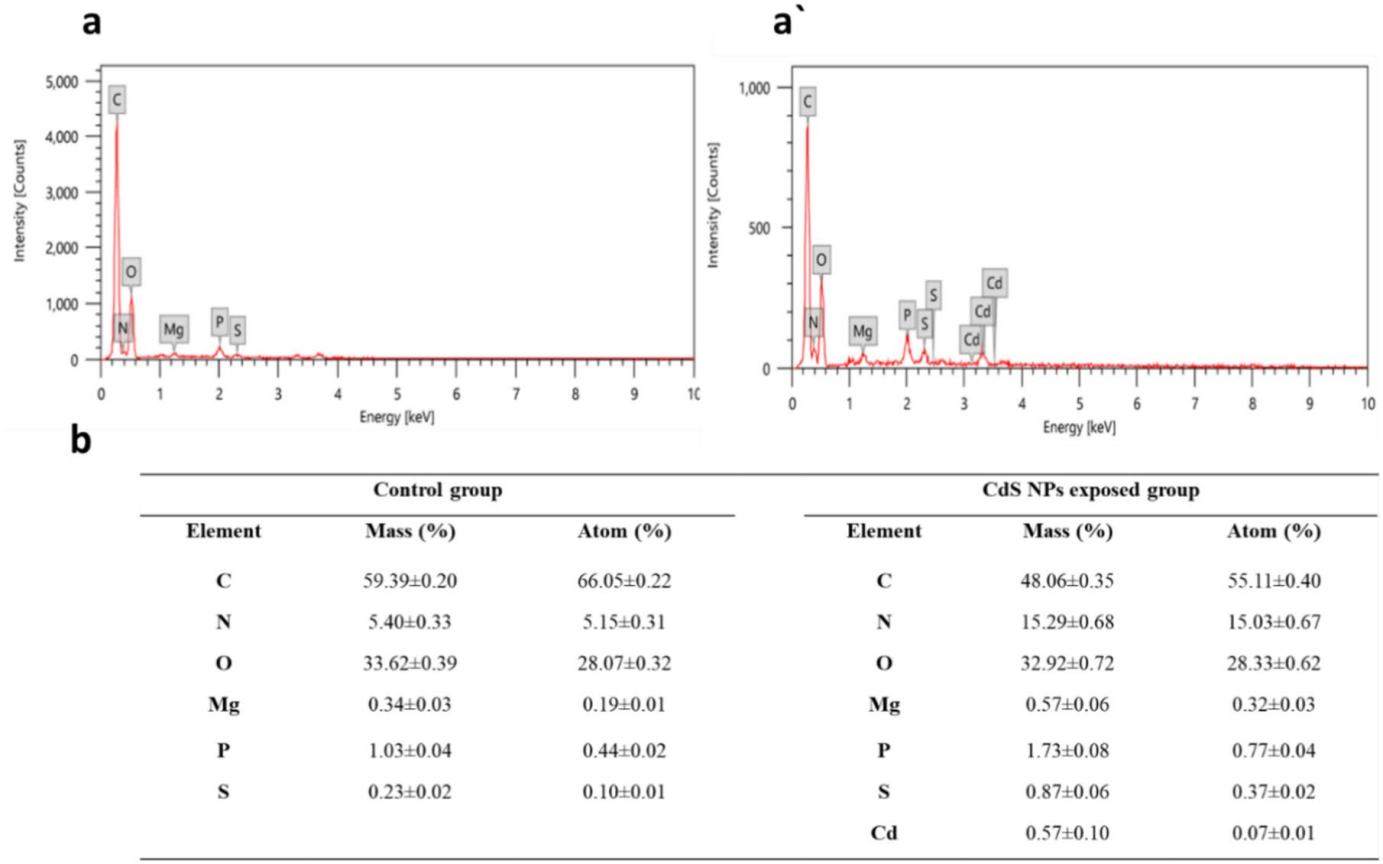
Illustrating the EDX analysis of both control (**a**) and treated larvae (**a'**) display EDX spectra of larval tissues from typical larvae and the CdS NPs-treated larvae, respectively, showcasing the presence of Cd and S in the CdS NPs-treated larvae. The EDX analysis (**b**) of larval tissues of *M. domestica* in response to CdS NP exposure, with results displayed as mean ± SEM.

Larval midgut tissues from the feeding group were treated with 123 μg/mL CdS NPs, and the control group underwent EDX analysis (Fig. 3). Control L3 midgut tissues showed elements like nitrogen (N), carbon (C), oxygen (O), phosphorus (P), magnesium (Mg), and sulfur (S). In the CdS NP-treated group, additional elements, including cadmium (Cd), were detected, indicating significant agglomeration with Cd content at 0.57 ± 0.10% and S at 0.87 ± 0.06%. Comparable amounts of other elements were observed in both control and treated larvae, as summarized in the Table (Fig. 3b).

### Cell viability

**Figure 4:**
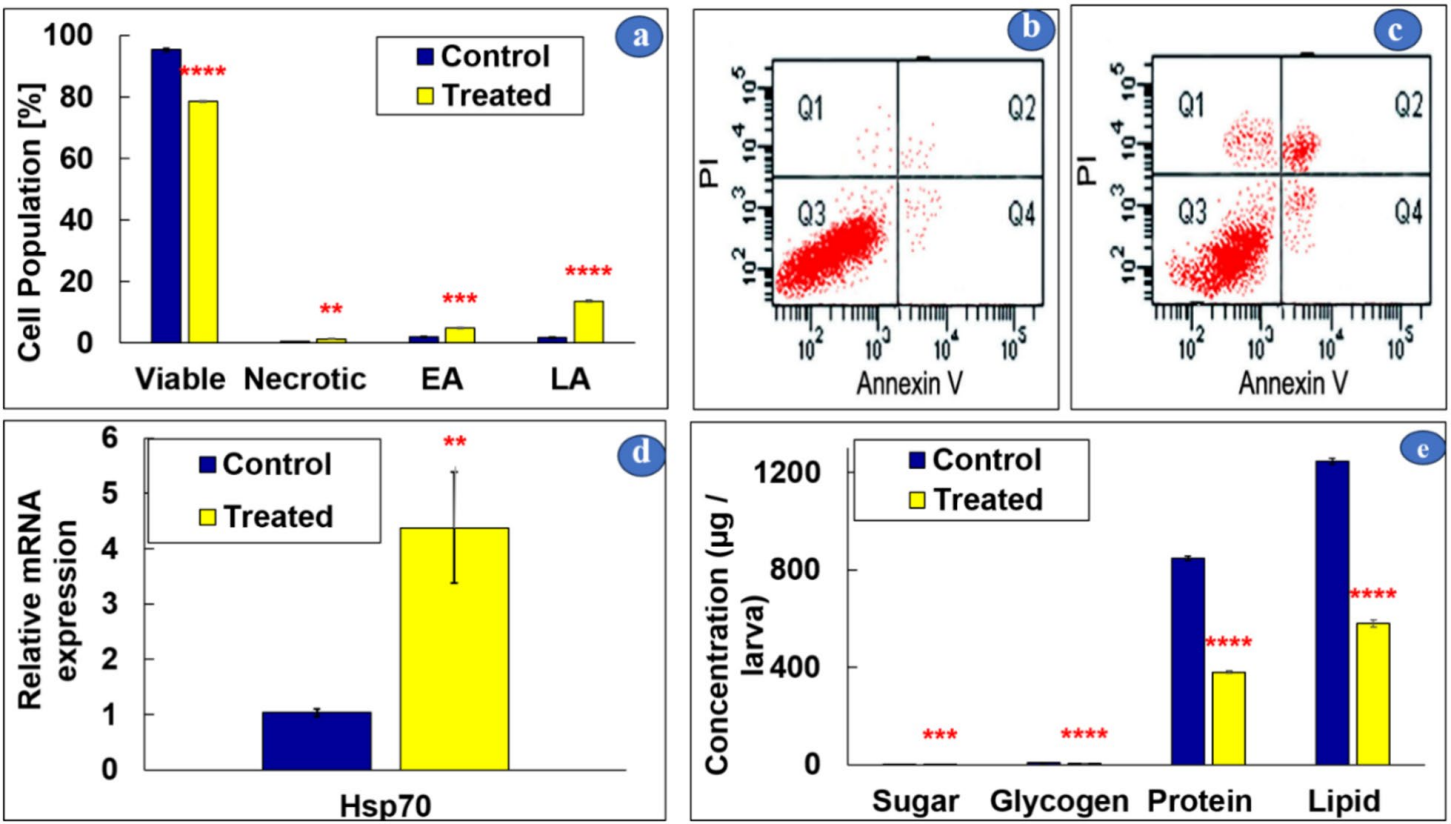
Flow cytometric analyses employing Annexin-V-FITC, conducted on *M. domestica* midgut larval tissues (**a**), with plots for the control (**b**) and CdS NPs-treated larvae at LC_50_ (123 μg/mL) through feeding (**c**). Relative expressions of HSP70 mRNA are presented in (**d**), and (**e**) illustrates the effect of CdS NPs on biochemical reserves. One-way ANOVA was used to evaluate the data, and Tukey's test was used for multiple comparisons. The stars above the bars indicate significant differences between groups (**p < 0.01, ***p < 0.001, ****p < 0.0001). Q1, Q2, Q3, and Q4 represent necrotic cells, late apoptotic cells (LA), viable cells, and early apoptotic cells (EA), respectively.

CdS NP-treated midgut larval tissues underwent cell viability assessment through annexin V-FITC flow cytometric analysis (Fig. 4a). The treated group at LC50 123 μg/mL exhibited a notable 78% decrease in living cell count and a significant increase in apoptotic cells compared to control larvae. In the control group, 0.6% exhibited signs of necrosis, while the CdS NP-treated group had approximately 1.4%. Specifically, 4.9% of CdS NP-treated larvae showed cells in early apoptosis, compared to the control group’s 2%. These differences were statistically significant, as illustrated in Fig. 4a. The flow cytometry graphs derived from the Annexin V-FITC assay (Fig. 4b and c) effectively represent necrosis, late apoptosis, viability, and early apoptosis (displayed across four quadrants, Q1–Q4). These graphical observations align with the results typically seen in the statistical data for both the control and treated groups, respectively.

### HSP70 gene expression

The study aimed to investigate the impact of LC_50_ CdS NPs exposure through feeding on the expression of HSP70 in the midgut of *M. domestica* L3, as depicted in (Fig. 4d). Compared to the control larvae, the group treated with LC_50_ 123 μg/mL CdS NPs exhibited a significant upregulation in the expression of the HSP70 gene.

### Effect of CdS NPs on biochemical components

Significant impacts were observed on the total body sugar, lipid, glycogen, and protein of L3 at the LC_50_ concentrations of CdS NPs. After 24 h of treatment with CdS NPs at the LC_50_ concentration, there was a significant and drastic reduction in the measures of larval body biochemical components, as illustrated in Fig. 4e.

### Scanning *electron* microscopy (SEM)

**Figure 5:**
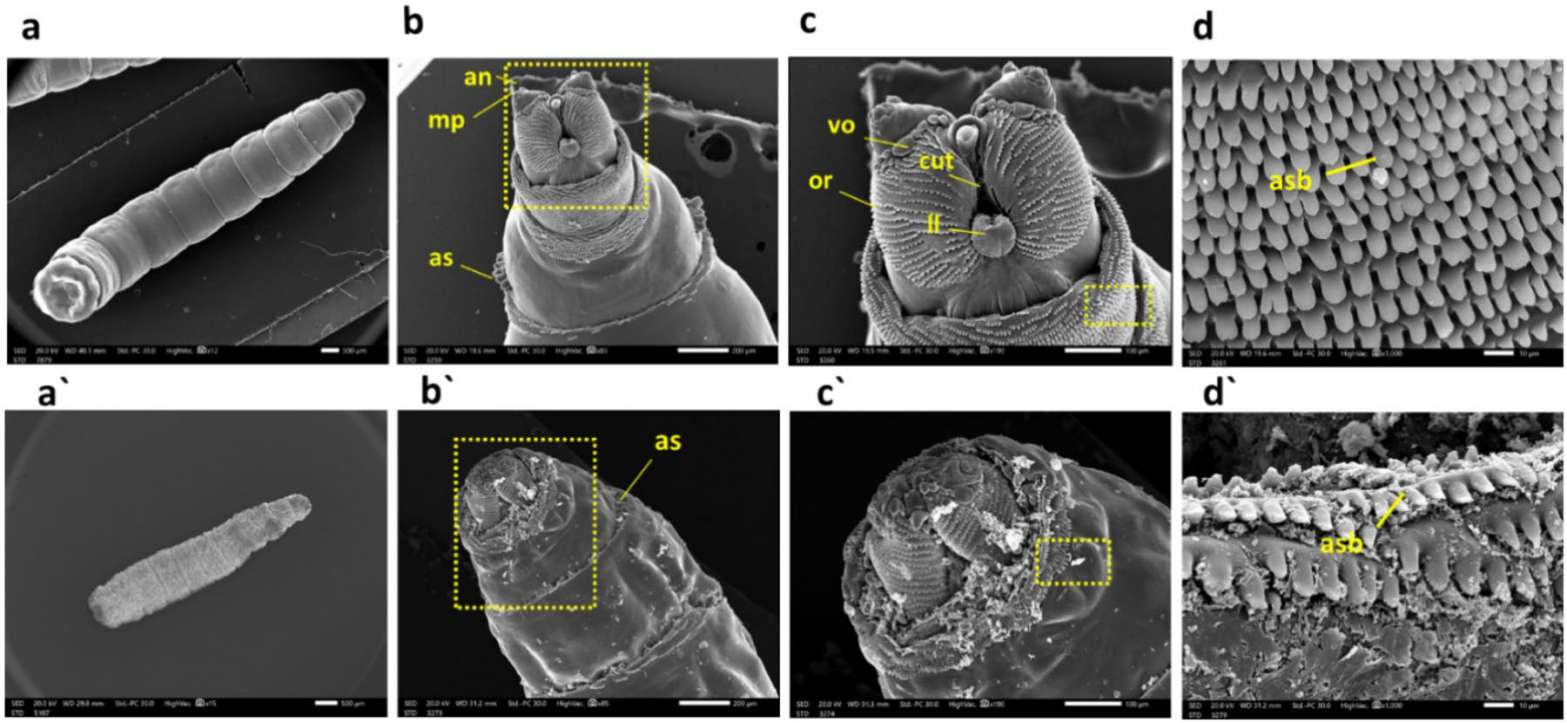
*M. domestica* L3 scanning electron photomicrographs: (**a**) Displays the control larva, while (**a'**) illustrates a shrunken larva from the CdS NPs-treated group. Moving to (**b**), it shows the anterior end of the 3rd larval instar with standard body details, including anterior spiracles (as), maxillary palpus (mp), and antennal complex (an). In contrast, (**b'**) depicts the anterior end of a larva treated with CdS NPs, indicating the degeneration of the antennal complex, maxillary palpus, and anterior spiracle. (**c**) Presents a higher magnification of the inset in (**b**), showing the control facial mask with details of cutaneous teeth (cut), oral ridges (or), labial lobe (ll), and ventral organ (vo). Meanwhile, (**c'**) shows a higher magnification of the inset in (**b'**), displaying the anterior end of the treated larva with extreme deformation of the antenna, maxillary palpus, and labial lobe. Finally, (**d**) illustrates a higher magnification of the inset in (**c**) of the control larva with an organized anterior spinose band (asb), and (**d'**) depicts a higher magnification of the inset in (**c'**) of the treated group with an unorganized, damaged anterior spinose band. Abbreviations: as = anterior spiracles; mp = maxillary palpus; an = antennal complex; cut = cutaneous teeth; or = oral ridges; ll = labial lobe; vo = ventral organ; s = spinules.

We used a scanning electron microscope to investigate the morphological damage inflicted on treated larvae versus the control group. The SEM micrograph analysis of the control group revealed regular smooth bodies and a typical appearance of the posterior end. In contrast, exposure to CdS NPs through the feeding method resulted in a significantly shrunken cuticle (Fig. 5a, a'). Furthermore, the treated larvae exhibited a distorted anterior end with a reduced-size maxillary palpus and antennal complex, along with smaller, distorted anterior spiracles versus the control larvae (Fig. 5b, b'). Notably, numerous changes in the facial mask were observed in CdS NP-treated larvae, particularly the degeneration of cutaneous teeth, oral ridges, labial lobe (ll), and ventral organ (vo) in comparison to the control larvae (Fig. 5c, c'). The anterior spinose band of preserved larvae was visibly disorganized and damaged, unlike typical larvae (Fig. 5d, d').

**Figure 6:**
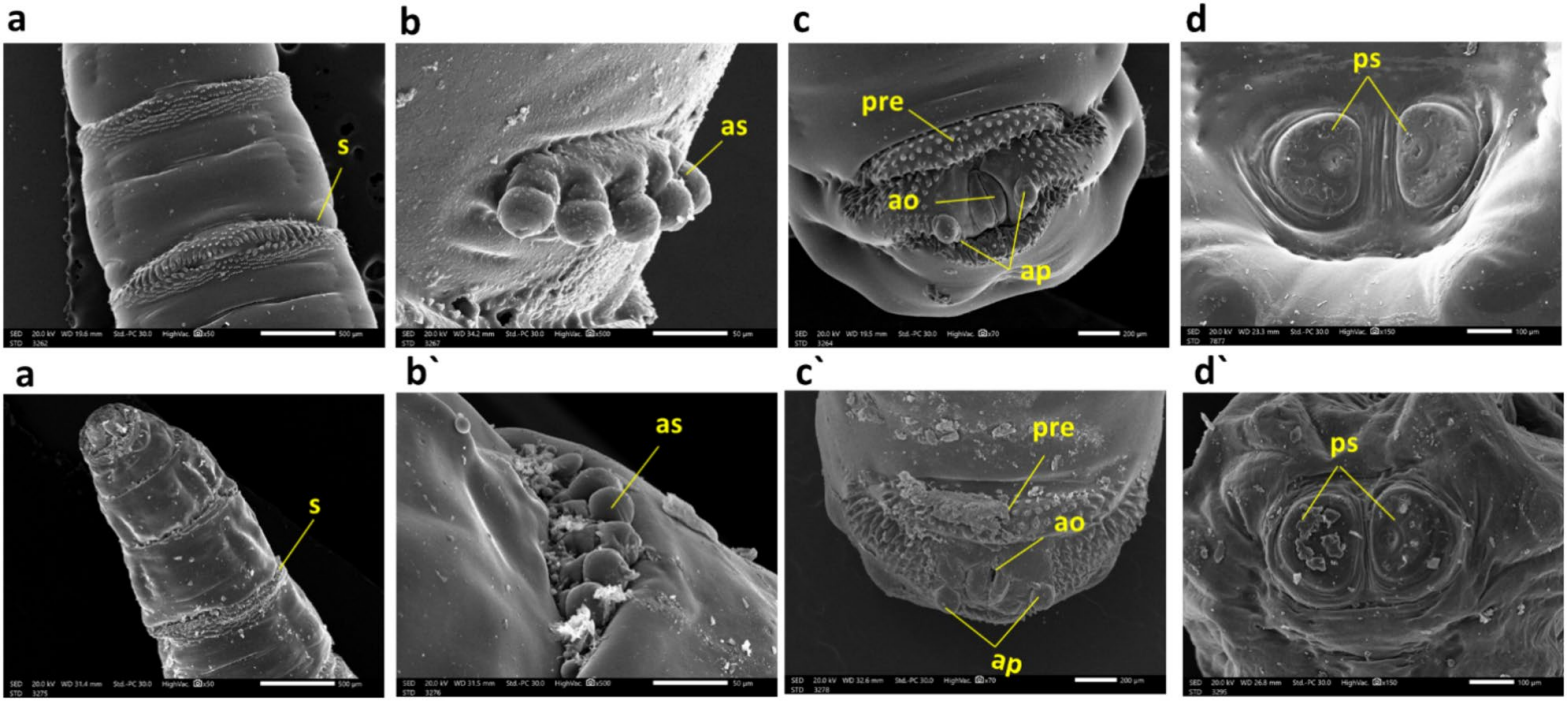
*M. domestica* L3 scanning electron photomicrographs: (**a**) depict the control group with normal intersegmental spinules, while (**a'**) illustrates the degeneration of intersegmental spinules in the treated group. In (**b**), a typical anterior spiracle is presented, and (**b'**) shows the anterior end of a larva treated with degeneration of the anterior spiracle. Moving to (**c**), the control larva's anal division is observed, noting the anal opening (ao), pre-anal welt (pre), and anal papillae (ap). In contrast, (**c'**) illustrates the posterior end of a larva treated with CdS NPs, indicating severe degeneration in the anal division. Finally, (**d**) displays the posterior end of the control larva with typical posterior spiracles (ps), while (**d'**) shows the posterior end of the larva treated with CdS NPs, revealing severe damage to the posterior spiracles. Abbreviations: s = spinules; as = Anterior spiracle; ao = anal opening; ap = anal papillae; pre = pre-anal welt; ps = posterior spiracles.

Furthermore, the ultrastructural examination of the control group showed intersegmental spines presenting a typical appearance contrasting with the treated group (Fig. 6a, a'). The treated larvae exhibited degeneration of the anterior spiracle, contrasting with the control (Fig. 6b, b'). The posterior end of larvae treated with CdS NPs showed severe degeneration in the anal opening, anal papillae, and pre-anal welt (Fig. 6c, c'). The spiracles of the posterior respiratory system showed extreme impairment contrasted with typical larvae (Fig. 6d, d').

**Figure 7:**
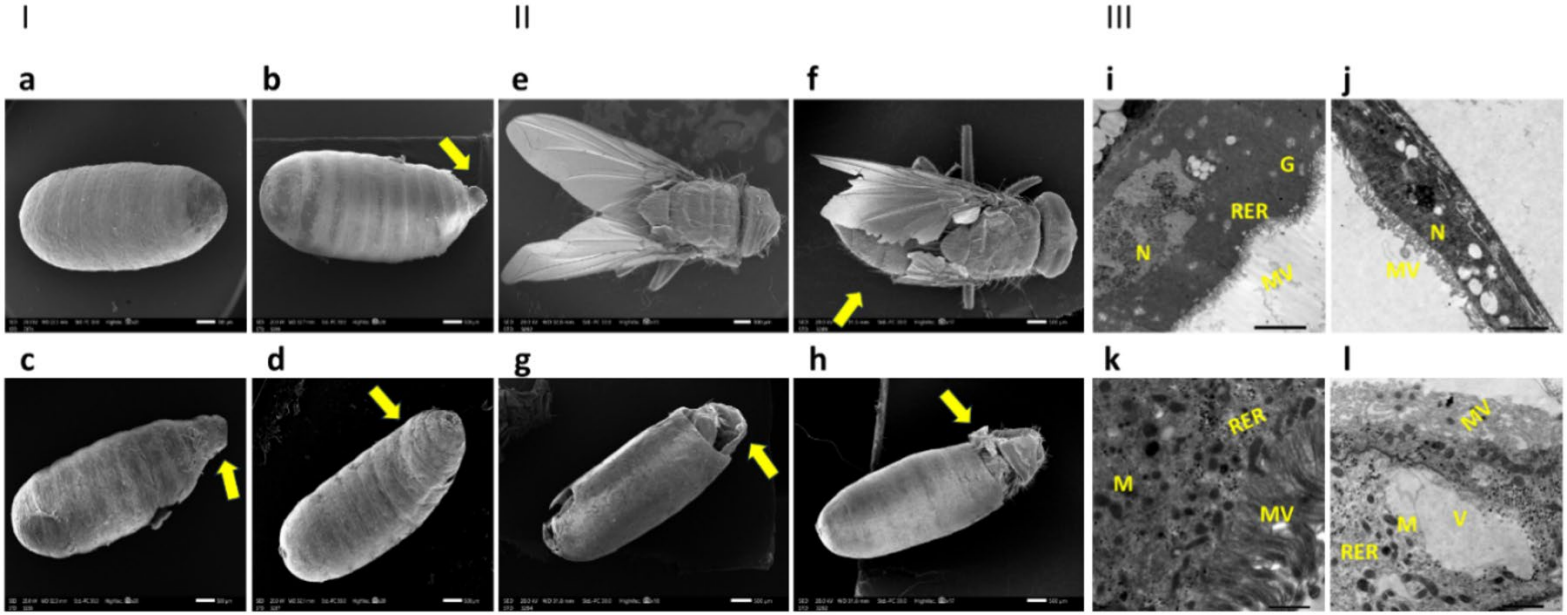
Part(I) arrow shows *M. domestica* pupae, with (**a**) representing the normal control appearance compared to treated pupae with an LC_50_ of CdS NPs through the feeding method, exhibiting aberrant anomalies demonstrated as (**b**, **c**) abnormal appendages of pupae, or another curved pupa (**d**). Part (II) arrow presents the adult housefly with (**e**) representing a typical appearance and (**f**) a distorted adult treated with the LC_50_ of CdS NPs through the feeding method, resulting in the adult being unable to fly. It also illustrates emergence with only the head region from the capsule (**g**) and emergence with only the head and thorax region from the capsule (**h**). Part (III) displays the transmission electron micrograph of midgut tissues of *M. domestica* L3: (**i**) Control L3 midgut details, including the normal nucleus (N), microvilli (MV), rough endoplasmic complex (RER), and Golgi complex (G). (**j**) Shows L3 midgut treated with the LC_50_ of CdS NPs through the feeding method. Note the extreme distortion of the nucleus and microvilli. (**k**) Presents the control L3 with well-organized microvilli, mitochondria (M), and rough endoplasmic complex. (**l**) Presents the treated group with damaged microvilli, abnormal rough endoplasmic complex combined with vacuolation (V) in the cytoplasm, and malformed mitochondria. Key: N = nucleus; MV = microvilli; RER = rough endoplasmic complex; G = Golgi complex; V = vacuolated cytoplasm.

Larvae capable of transforming into pupae often exhibited deformations compared to the control pupa with normal pupal spiracles (Fig. 7a). Abnormal appendages were observed in treated pupae (Fig. 7b, c), and several pupae showed a curved shape (Fig. 7d). Treated pupae that successfully transformed into adults were mostly deformed and unable to fly (Fig. 7f) compared to the control adults, which were able to fly (Fig. 7e). It was also noted that most adults appeared with the emergence of the head region only from the capsule (Fig. 7g) or the emergence of the head and thorax region from the capsule (Fig. 7h).

#### Ultrastructural investigations

The larval midgut ultrastructure was assessed using TEM, revealing distinct characteristics. The typical larval midgut appeared condensed and exhibited an abundance of elongated microvilli resembling a brush boundary. The cell cytoplasm was compact and well-ordered, incorporating a regular nucleus, microvilli, rough endoplasmic reticulum, and Golgi complex.However, when subjected to a lethal dose of 123 µg/mL of CdS NPs through the feeding method, the L3 experienced substantial harm in their midgut (Fig. 7i, j). Cytoplasmic lysis was noted in midgut cells of the treated group, and noticeable deformities were observed in the rough endoplasmic complex, nucleus, microvilli, and mitochondria, which decreased in number. Additionally, there was an increase in the number of vacuoles compared to typical L3 (Fig. 7k, l). These findings indicate that the lethal dose of CdS NPs caused significant structural alterations in the larval midgut, affecting various cellular components and organelles.

## Discussion

The present study concentrated on the environmentally friendly method of green CdS NPs synthesis^70,71^. CdS NPs were synthesized using *Agaricus bisporus* and assessed for their larvicidal efficacy against L3 of common houseflies. The observed blue shift in the absorption band edge, indicating a larger band gap in CdS NPs compared to micrometer-sized particles, suggests the quantum size effect. This well-documented phenomenon in nanomaterials alters electronic properties due to reduced dimensions. The results infer that CdS nanoparticles confined within pore channels have an estimated average diameter of less than 10 nm, supported by previous findings^65^.

The FTIR analysis of CdS NPs revealed distinct peaks: 1618.29 cm^−1^ indicated carbonyl stretching vibrations linked to amide-I in proteins from mushroom plant-derived constituents. Peaks at 1399.60, 1000–1120, and 620.30 cm^−1^ offered insights into additional molecular bonds, emphasizing comprehensive CdS NP characterization. These findings, supported by existing literature^67,68^, contribute to a thorough understanding of the nanoparticle composition and hold significance for potential applications.

Distinct XRD peaks align with the expected hexagonal CdS structure, but broadening from residual plant constituents is a key consideration. Despite this, the absence of impurity peaks underscores high purity, which is crucial for reliable analyses and diverse applications. Additionally, the negative zeta potential of − 19.5 indicates enhanced electrostatic repulsion among CdS NPs, which is crucial for preventing coagulation and ensuring dispersion stability. This stability, vital for diverse nanotechnology and materials science applications, is imperative for effective utilization in entomology and other fields. SEM images show both spherical particles and aggregations in CdS NPs. Confirmation and determination of average particle size (5–8 nm) through TEM images provide precise insights consistent with UV–Vis spectrum results, ensuring reliability and internal consistency in CdS NPs characterization.

In our study, we investigated the green CdS NP's biological activity versus the L3 stage of house flies at various concentrations of NPs (75, 100, 125, 150, 175, and 200 µg/mL). Our results indicated that the LC_50_ value for the feeding method was 123 μg/mL (Fig. 2a), while it was 138 μg/mL for the dipping method ((Fig. 2a'). These findings align with an earlier finding by^50^, where they assessed the larvicidal effects of magnetite nanoparticles (Fe_3_O_4_ NPs) on *M. domestica* L3 using contact and feeding methods at six concentrations of Fe_3_O_4_ NPs (15, 30, 45, 60, 75, and 90 μg/mL) in the feeding method, with the reported LC_50_ value being 60 μg/mL. Similarly, The LC_50_ value for the dipping method was found to be 75 μg/mL.

Specific elements were present in the *M. domestica* L3 midgut tissues from the control group (Fig. 3a), according to the EDX analysis. However, in the group treated with 123 μg/mL of CdS NPs, the presence of Cd along with the previously detected elements was observed (Fig. 3a', b), which supports prior findings by Mir et al.^72^ regarding Zinc oxide nanoparticles (ZnO NPs) accumulation in various tissues of *Bombyx mori*.

Assessment of cell viability using cytometric Annexin V estimation revealed that CdS NPs-treated larvae of *M. domestica* at LC_50_ (123 μg/mL) through feeding exhibited a considerable decrease in the number of viable cells and an increase in the prevelance of apoptotic cells as illustrated in Fig. 4c. Similar results are consistent with earlier findings by Dabour et al.^73^), in which flow cytometry analysis confirmed a high percentage of together necrosis and apoptosis in bees revealed to sublethal concentrations of Cadmium Oxide nanoparticles (CdO NPs) in sugar syrup. The observed increase in apoptosis, a programmed and regulated cell death process, reflects an attempt to preserve cellular homeostasis and can indirectly indicate an elevation in cellular abnormalities in response to exposure to CdS NPs.

Stressed organisms employ various defense mechanisms to mitigate the harmful effects of toxins, as stated by Tarnawska et al.^74^). El-Samad et al.^75^) previously conveyed a significant induction of HSPs through 1.5 toward fourfold, which can enhance cell survival and resistance to oxidative stress-induced damage in insects, as observed in our study with *M. domestica* L3 exposed to CdS NPs (Fig. 4d). Insects with elevated levels of HSPs mRNA have a higher likelihood of surviving and displaying increased resistance to damage caused by oxidative stress, as demonstrated in a study by Dong et al.^76^). The HSP70 family of proteins plays a crucial role in rescuing misfolded and accumulated proteins by unfolding them, allowing proteins to resume their proper folding pathway^77^. The significant upregulation of HSP70 gene expression was observed in *M. domestica* L3 studied with CdS NPs, which contrasted with the control group, indicating a potential protective response against stress. Comparable results were stated by Kheirallah et al.^78^) in the Nickel Oxide nanoparticles (NiO NPs) treated group, where HSPs mRNA was upregulated and prevented the aggregation of damaged proteins, which could cause severe harm to stressed cells.

The existence of nutritional reserves like lipids, sugars, and proteins plays a critical role in insects' growth and development. Consequently, we investigated the effects of CdS NPs on these essential metabolites in house fly L3. Larvae exposure to CdS NPs at LC_50_ concentrations triggered a substantial lessening in the overall total body glycogen, sugar, lipids, and protein levels (Fig. 4e). These findings align with the observations made by Chintalchere et al.^2^), who documented a decrease in protein, lipid, and carbohydrate concentrations following exposure to tea tree essential oils (TTEOs) and lemongrass (LG). However, a decline in both sugar and glycogen content observed later in treatment with CdS NPs may be attributed to their contribution to resisting the chemical stress induced via the assessed compounds^46^. Consequently, the disruption of metabolic processes caused by Cd-treated larvae likely contributed to larval mortality^79^.

The decline in protein content observed following exposure to CdS NPs might be attributed to protein breakdown into component amino acids, which may hinder the growth and development of insects (Fig. 4e). This finding aligns with the observations made by^80^. Similarly, the decrease in sugar content observed in treated house fly L3 may be indicative of the intensified carbohydrate resource utilization under stress conditions, serving to maintain vital physiological metabolic functions. This interpretation is in arrangement with the assumptions reached by^81^, who documented a decrease in lipids in larvae of *Agrotis ipsilon* following treatment with Chitosan and Silver nanoparticles (AgNPs).

In our current study, a morphological examination using SEM was conducted on the typical L3 of *M. domestica* (Fig. 5a). The observed morphological features closely resembled those reported by other authors who have studied similar fly larvae^82,83^. These previous studies also documented the presence of well-developed cephalic sensory organs and mouthparts in the larvae. Additionally, our examination encompassed the ultrastructure of various sensory organs, including the antenna and maxillary palp, mouthparts, anterior (Fig. 5b) and posterior spiracles (Fig. 6d), and intersegmental spines (Fig. 6a).

Lethal dose-treated larvae showed significant surface deformations than the untreated ones. SEM examination of treated *M. domestica* L3 showed a dense condensed body with a distorted anterior end structure, which was much smaller in size, with minute oral hooks, abnormal shrunken anterior spiracles (Fig. 5a'–b'), and anomalies in the structure of both anterior and posterior spiracles (Fig. 6b' and d'). Our results approve the opinion of El-Ashram et al.^26^), who observed similar effects in *Chrysomya albiceps* larvae that were reared on Aluminum phosphide (AlP) intoxicated rabbits. They reported the presence of a densely condensed body near the damaged anterior end, along with tiny oral hooks, as well as abnormal spiracles. Additionally, the posterior end exhibited distortions and damaged respiratory spiracles. Also, the present outcomes are coherent with those reached by Subaharan et al.^84^), who documented the occurrence of larval cuticle shrinkage, changes in the intersegmental area, along the spinous cells evolution in house fly larvae treated with *Piper betle* L. essential oil. Additionally, our result is similar to Elshehaby et al.^85^), who found abnormally attached tiny respiratory spiracles and a distorted posterior end with posterior respiratory spiracles hypogenesis in L3 of *Chrysomya albiceps* treated with tramadol. Earlier Kumar et al.^86^) noted that the larvae of houseflies treated with *Eucalyptus globulus* showed distortions in their integument, else intersegmental spines.

Our results also revealed that treated larvae were able to pupate, showed curved pupae, and had abnormal appendages (Fig. 7a–d), which aligns with the results of Toto et al.^50^), similar disruptions in the pupal development of *M. domestica* due to Fe_3_O_4_ NPs exposure.

The deceased adult flies exhibited morphological abnormalities, such as the inability to fly and partially emerged bodies (Fig. 7e–h). Also, Abdel‐Baki et al.^87^ found that lesser concentrations of trans-anethole and trans-anethole fennel oil substantially influenced pupal survival, resulting in adults with underdeveloped wings or incomplete emergence.

The electron micrographs revealed some ultrastructural variations in the organelles of cytoplasm specifically associated with microvilli interruption, vacuolization of cytoplasm and overall disorganization, mitochondria alteration, and endoplasmic reticulum distortion (Fig. 7i–l), which show severe degeneration of larval midgut. These ultrastructural variations resemble the effects described by^88^, who described the cause of *Brevibacillus laterosporus* ingestion on *M. domestica* larval midgut ultrastructure. Interestingly^55^, Dabour et al.^73^ reported that CdO NPs-treated bees revealed nuclear content lysis, proteolysis of cytoplasm, Microvilli appeared detached, and RER membranes were less, else mitochondrial elongation was detected.

## Conclusion

Our study showed that green-synthesized CdS NPs from A. bisporus effectively controlled M. domestica larvae, with feeding treatment proving more potent than dipping. Treated larvae displayed morphological deformities, resulting in elevated mortality across larval, pupal, and adult stages. The correlation between Cd accumulation, apoptotic cells, stress biomarkers, and biochemical changes in the larval midgut emphasizes the harmful effects of CdS NPs. Additionally, exposure induced noticeable ultrastructural changes in midgut tissues, highlighting the need for future investigations. The biogenic synthesis of CdS NPs is an eco-friendly and innovative approach leveraging natural extracts' inherent capabilities for controlled growth, guided by biomolecules like proteins and enzymes, making it environmentally conscious, cost-effective, and uniquely shaped by natural source's phytochemical composition. The cost of producing CdS NPs via this green synthesis method is significantly lower than conventional methods due to the elimination of toxic chemicals and the use of readily available materials, such as mushroom extracts, which are both cost-effective and environmentally sustainable. Future research should focus on scaling up the green synthesis process to industrial levels, ensuring consistency and quality, and exploring the long-term environmental impacts and broader applications in other environmental and industrial contexts.

## Supplementary Information

Supplementary Information.

## Data Availability

The data sets used and analyzed during the current study are available from the corresponding author on reasonable request.
