# Supplementary material for: Assessing the toxicity of green Agaricus bisporus-based Cadmium Sulfide nanoparticles on Musca domestica as a biological model
Source: Sci Rep. 2024 Sep 14;14:21519. doi: 10.1038/s41598-024-70060-y (PMC11401898; doi:10.1038/s41598-024-70060-y)
Supplement: Supplementary file 1 — Supplementary Information. [file 41598_2024_70060_MOESM1_ESM.pdf]

Supplementary Material

Assessing the Toxicity of green *Agaricus bisporus*-based Cadmium Sulfide nanoparticles on *Musca domestica* as a biological model

Hanan I. Elhenawy [a], Noura A. Toto [b], Abdelazeem S. Eltaweil [c,d], Hussein K. Hussein [a], Maria Augustyniak [e], Lamia M. El-Samad [a,*]

[a] Department of Zoology, Faculty of Science, Alexandria University, Egypt

[b] Department of Zoology, Faculty of Science, Damanhour University, Egypt

[c] Department of Engineering, College of Engineering and Technology, University of Technology and Applied Sciences, Sultanate of Oman

[d] Chemistry Department, Faculty of Science, Alexandria University, Alexandria, Egypt

[e] Institute of Biology, Biotechnology and Environmental Protection, Faculty of Natural Sciences, University of Silesia in Katowice, Bankowa 9, 40-007 Katowice, Poland

*Corresponding authors

Lamia Mostafa Abd ElSamad: lamya.moustafa@alexu.edu.eg

Text S1

Characterization of CdS NPs

CdS NPs was characterized by UV-Vis spectroscopy to examine their optical properties, However, x-ray diffractometer (XRD; MAC Science M03XHF) to examine their crystal phases, Fourier transform infrared Spectrometer (FTIR- Shimadzu-8400S) to recognize the functional groups of the samples, scanning electron microscope (SEM; S4800, Hitachi) to explore the morphology of the samples. Transmission electron microscopy (TEM) was used to determine the particle size of CdS NPs (JEOL-JEM 2100 instrument operating at 120 keV). Zeta potential analyzer (ZP- Malvern, UK) used to examine the surface charge of the composite.

Text S2

Effect of CdS NPs on glycogen, sugar, and lipid contents

The effects of CdS NPs treatment on the larval body were examined by subjecting a group of twenty-third instar larvae to the LC50 of test NPs. Following the treatment, the larvae underwent anesthesia, and their biochemical components, such as the contents of lipids, total sugar, and glycogen, were evaluated. Then, the larval bodies were homogenized in a solution containing 0.2 mL of sodium sulfate (w/v). Following homogenization, 0.8 mL of methanol in chloroform (1:1, v/v) was added. The mixture was centrifuged for two minutes at 3000 rpm, yielding a supernatant. The amount of glycogen was estimated using the Anthrone procedure [1]. To form two layers, the pellet from the centrifugation step was

resuspended in 3 mL of distilled water (dH2O) and centrifuged again for two minutes at 3000 rpm. The Van Handel and Day method [2] was used to analyze the sugar content in the upper aqueous layer. The lower layer, primarily containing lipid-containing chloroform, was combined with 0.2 mL H2SO4 and subjected to a 10-minute heating at 90° C. The resulting color change was measured at 625 nm using an Agilent Technologies Carry 60 spectrophotometer made in the USA after adding 5 mL of Vanillin reagent. Glucose served as the standard for sugar and glycogen content determination, while soybean oil was the standard for lipids. The Bradford assay was used to determine the body's total protein level [3,4], with BSA as the standard. The values for the biochemical components were expressed in micrograms (µg) per larvae, based on the standards used.

References:

1 Chintalchere, J., Dar, M., Raut, K. & Pandit, R. Bioefficacy of Lemongrass and Tea Tree Essential Oils Against House Fly, Musca domestica. *Proceedings of the National Academy of Sciences, India Section B: Biological Sciences* **91**, doi:10.1007/s40011-020-01220-z (2021).

2 Chintalchere, J., Dar, M. & Pandit, R. Biocontrol efficacy of bay essential oil against housefly, Musca domestica (Diptera: Muscidae). *The Journal of Basic and Applied Zoology* **81**, doi:10.1186/s41936-020-0138-7 (2020).

3 Poodts, J. *et al.* Improved Expression of SARS-CoV-2 Spike RBD Using the Insect Cell-Baculovirus System. *Viruses* **14**, 2794 (2022).

4 Bradford, M. M. A rapid and sensitive method for the quantitation of microgram quantities of protein utilizing the principle of protein-dye binding. *Analytical Biochemistry* **72**, 248-254, doi:https://doi.org/10.1016/0003-2697(76)90527-3 (1976).
